# Micropatterning of cells via adjusting surface wettability using plasma treatment and graphene oxide deposition

**DOI:** 10.1371/journal.pone.0269914

**Published:** 2022-06-16

**Authors:** Nosayba Al-Azzam, Anas Alazzam

**Affiliations:** 1 Department of Physiology and Biochemistry, Jordan University of Science and Technology, Irbid, Jordan; 2 System on Chip Lab, Department of Mechanical Engineering, Khalifa University, Abu Dhabi, UAE; The University of Tokyo, JAPAN

## Abstract

The wettability of a polymer surface plays a critical role in cell-cell interaction and behavior. The degree to which a surface is hydrophobic or hydrophilic affects the adhesion and behavior of cells. Two distinct techniques for patterning the surface wettability of a Cyclic Olefin Copolymer (COC) substrate were developed and investigated in this article for the purpose of patterning cell growth. These include oxygen plasma treatment and graphene oxide (GO) coating to alter the wettability of the COC substrate and create hydrophilic patterned regions on a hydrophobic surface. When the two techniques are compared, patterning the surface of COC using GO film results in a more stable wettability over time and increases the roughness of the patterned area. Interestingly, both developed techniques were effective at patterning the COC surface’s wettability, which modulated cell adhesion and resulted in micropatterning of cell growth. The novel methods described herein can be used in the fields of cell and tissue culture as well as in the development of new biological assays.

## Introduction

The wettability of a polymer surface plays a critical role in determining how cells behave. The surface functional groups and roughness of a polymer surface affect its wettability [1]. Cell adhesion is largely determined by the wettability of the surface, rather than by the surface functional groups, surface density, or cell type [2]. The wettability of a surface controls the adhesion of cellular proteins and thus has an effect on cell behavior [3]. The most common way to determine a polymer surface’s wettability is to measure its water contact angle. Typically, the majority of mammalian cells prefer to adhere to and grow on surfaces that are moderately hydrophilic but not superhydrophilic (contact angle less than 5°) or superhydrophobic (contact angle greater than 150°) [1,4]. Cells adhered best to surfaces with contact angles of between 40° and 70° [2]. While hydrophilic surfaces can promote cell differentiation, superhydrophobic surfaces can alter the conformation of fibronectin, thereby impairing cell adhesion [5]. For example, hydrophilic surfaces such as clean glass and aminopropylsilane (APS) promote fibroblast adhesion and proliferation more than hydrophobic surfaces such as polylactate (PL) and silicone (SI) [6].

Polydimethylsiloxane (PDMS) is a widely used material in biological microfluidics and microsystems. This material was recently proposed as a good biomaterial for the production of extracellular matrix (ECM) patterns; however, its hydrophobicity prevents it from being used in cell-based methodologies [7]. The wettability of PDMS was temporarily improved by plasma treatment, with the limitation that its hydrophobicity returned after a few hours of air exposure. Interestingly, due to its increased wettability, a novel PDMS-type (X-PDMS) demonstrated better compatibility with cell growth [8].

Plasma is made up of extremely excited molecular, atomic, ionic, and radical species. When gases are excited into energetic states, plasma is formed [9]. Using various reactive gases such as air, NH_3_, CO_2_, N_2_, and O_2_, plasma can introduce polar functional groups such as hydroxyl, carboxyl, and amino groups onto various polymer surfaces. As a result, plasma treatment increases hydroxylation and carboxylation on treated surfaces, making the surface more hydrophilic and increasing cell adhesion to various biomaterials. The hydrophilic nature of treated surfaces typically promotes adsorption of some ECM biomolecules such as fibronectin and collagen, which then interact with cellular integrin receptors, resulting in increased cellular binding [10]. Water vapor plasma discharge treatment can be used to modify a wide range of polymer surfaces. Polyethylene, polypropylene, polystyrene, polyethylene terephthalate, and poly (methyl) methacrylate surfaces are examples. The water vapor plasma treatment increases the wettability of polymer surfaces and introduces a large number of hydroxyl functional groups. Water vapor plasma-treated polymers have thus been linked to improved cell adhesion, spreading, and growth properties [11]. On the other hand, plasma glow discharge treatment of polymer surfaces modifies the wettability of all surfaces in proportion to the plasma treatment duration [12]. Atmospheric pressure air plasma stenciling methods were used to create hydrophilic regions on hydrophobic PDMS, methylated glass, and polystyrene surfaces, which resulted in the successful micropatterning of various cell types. This method did not employ adhesion molecules or cell repellent patterning steps, which greatly simplifies the process of achieving highly effective, reproducible, and long-term cell patterns [13].

Graphene is a single layer of carbon atoms in a honeycomb structure, with each carbon atom covalently bound to three neighbors [14]. Graphene has unique electrochemical properties such as very high hydrophobicity, high thermal conductivity, high current carrying capacity, chemical inertness, and optical transmittance, which has sparked a lot of interest in a variety of fields [15]. The surface physicochemical properties of graphene, as well as its 2D allotropic structure, play an important role in various graphene-based materials that can be used in biomedical and bioelectronic applications [15]. Graphene can be synthesized in a variety of sizes, shapes, and chemical modifications, resulting in a variety of biological outcomes in research fields [16]. Graphene oxide (GO), an oxidized form of graphene, has recently gained potential importance in biological research, such as drug delivery and bio-analysis [17]. Functionalized GO nanosheets on a patterned gold surface, for example, were successful in isolating circulating tumor cells from blood samples of patients with pancreatic, breast, and lung cancer [18]. We recently developed a solution-based method for patterning GO thin films on transparent substrates that is simple and repeatable [19]. GO thin films were patterned on substrates using an adapted liftoff method in conjunction with plasma treatment of the substrate. The patterned GO films were successfully integrated into a microfluidic system, demonstrating their potential for use in a variety of lab-on-chip applications [19–22]. The hydrophilic GO can be patterned on the hydrophobic emerging material, cyclic olefin copolymer (COC). GO is made up of graphene sheets that have been decorated with oxygen-containing functional groups. Because oxygen functional groups have a negative charge, they can form hydrogen bonds, which increases the hydrophilicity of GO sheets. Many applications exist for the use of a patterned GO thin film with tunable hydrophilicity [21,22].

Graphene is a biocompatible material that is safe to use biologically because it does not cause an immune or sensitivity reaction and can be degraded *in vivo* by macrophages [23]. With a dose of less than 20 μg/ml, graphene oxides showed no toxicity to human fibroblast cells. However, cytotoxic effects such as decreased cell adhesion, induction of cell apoptosis, and entry into lysosomes, mitochondrion, endoplasm, and cell nucleus have been observed in response to doses greater than 50 μg/ml [24]. GO stimulates both bacterial and mammalian cell adhesion and proliferation [25]. GO films deposited from a 2 mg/mL GO dispersion can be used as a cell culture substratum for adipose stem cells. When human adipose stem cells are grown on GO-coated glass rather than non-coated glass, their adhesion, proliferation, and differentiation are increased [26]. Furthermore, through molecular interactions with various chemical inducers, GO can modulate stem cell adhesion, growth, and differentiation [27].

Efficient cell patterning or cell printing necessitates the creation of cell adherent regions that are immediately adjacent to cell repellent regions. These different areas must remain stable during the cell culture process [13]. Cell adhesion is important for cell communication and regulation, as well as tissue development and maintenance. "Cell adhesion is the ability of a single cell to stick to another cell or an extracellular matrix (ECM)". When cultured *in vitro*, the majority of mammalian cells adhere strongly to the substrate. Cells adhere better to surfaces where they can form stronger chemical bonds [28]. Cells can interact and communicate with the ECM by producing three major signals: chemical, mechanical, and topographical, all of which can influence cell behavior [4]. A physicochemical cell-surface reaction occurs when cells attach to the surface of a certain material. Protein adsorbs to the surface material first and then mediates cell adhesion. Once adhered to the surface, cells release active molecules that promote ECM deposition, cell differentiation, and proliferation [29].

Previously published research on growing cells on silicon wafers coated with GO reported that the area covered by the cells and the quantity of cells for a given growth period in an incubator were significantly dependent on the hydrophilicity and oxygenated groups present in the GO and on the Si substrate [30]. Adjusting the wettability of common tissue culture substrates has been shown to allow cell micropatterning [31]. There are no previous investigations to the authors’ knowledge on micropatterning cell growth utilizing patterned surface wettability using GO. Additionally, there is no prior work on bioprinting on polymer surfaces using the cell as the fundamental unit of printing via surface wettability finetuning. As a result, this work investigates the capability of modulating cell attachment and growth over patterned plasma-treated as well as GO-coated COC substrates. Both techniques were used to control the growth of cells on the surface and pattern the wettability of polymer substrates. The newly reported methods for manipulating the wettability profile of the COC surface were successfully employed to print on the COC surface using the cell as the fundamental unit of printing. This is the first attempt to pattern the wettability of COC’s surface using GO and then utilize it to micropattern cells. In the future, this method could be used in tissue engineering and the development of novel biological assays.

## Methods

### GO and plasma patterning on COC surfaces

**Fig 1:**
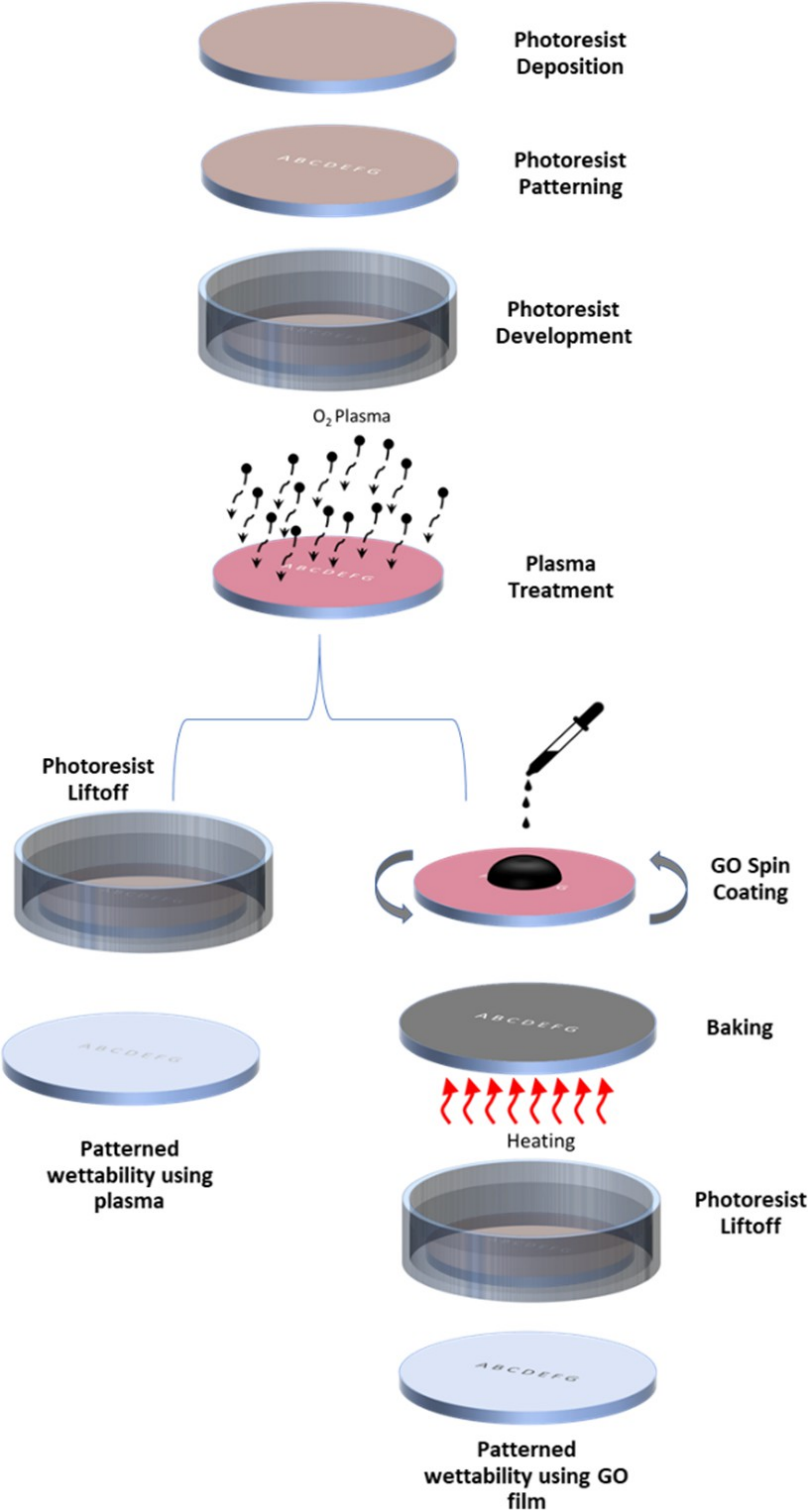
Schematic diagram illustrating the fabrication steps involved in patterning the COC wafer’s surface wettability using plasma and GO film.

Two distinct approaches to patterning the wettability of the COC substrate’s surface for cell growth patterning were investigated in order to develop a new method for printing with cells. **Fig 1** depicts a schematic diagram of the fabrication processes used to pattern the surface wettability of the COC surface using plasma and a GO film. Standard photolithography processes and oxygen plasma are used in the first approach to pattern the wettability of the COC surface. The substrate is first cleaned by sonication in isopropanol and water baths for five minutes each. The substrate is then dried with compressed nitrogen and baked at 70°C for ten minutes before a layer of photoresist (PR1813) is deposited using a spin coater (WS650Hzb-23NPP UD-3 from Laurell Technologies Corporation, North Wales, PA, USA). The photoresist layer is then baked and patterned with a photolithography system (Dilase 650 from KLOE, France). After that, the substrate is developed in a suitable developer and sonicated in deionized water (DI) before being dried with compressed nitrogen. After that, the substrate is treated with plasma (PDC-002 from Harrick Plasma, USA) for five minutes at a pressure of 700 mTorr and a power of 29.6 W to change the wettability of the exposed (patterned) area of the substrate. It was then sonicated in an Acetone bath to remove the photoresist layer. Following this stage, the exposed substrate is hydrophilic with a contact angle of about 20°, whereas the surface protected by the photoresist film is still hydrophobic with a contact angle of 110° [32]. In the second approach to controlling and patterning the wettability of the COC substrate, GO aqueous solution is uniformly deposited on the substrate using spin coating immediately after the substrate has been treated with plasma. The substrate is then baked at 70°C for ten minutes before being sonicated in an Acetone bath to remove the photoresist layer. Finally, the substrate is thoroughly washed in a DI water bath before being dried with compressed nitrogen. As a result, the hydrophilic GO-coated portion of the substrate has a contact angle of about 30° for GO concentration of 1 mg/ml.

### Cell culture

The MDA-MB-231 human breast cancer cells were cultured in RPMI media supplemented with 10% fetal bovine serum (FBS), 100 units/ml penicillin-streptomycin (Thermo Fisher Scientific), and maintained at 37°C in a humidified 5% CO2 atmosphere. The media was changed every other day, and the cells were trypsinized and split when they reached 80–90 percent confluence.

### Contact angle measurement

A contact angle goniometer was used to determine the contact angles between the COC surface and the DI sessile drops (L2004A1 from Ossila). Droplets of approximately 5 μl were placed gently on the surface, and the angle between the surface and the sessile drop was measured. The measurements were carried out ten times with different droplets on the COC wafer’s surface.

### Cell patterning

Cell printing via patterning of cell growth entailed seeding cells on plasma-treated or GO-coated COC substrates with no further surface modifications. COC substrates with patterned wettability were placed inside Petri dishes and sealed from the sides with PDMS. The dishes were then thoroughly washed in ethanol and DI water before being sterilized and disinfected with UV light. Cells were seeded in a 10 mL media containing 0.5×10^6^ cells and incubated for 24 hours at 37°C in a humidified 5% CO_2_ environment. The media was then aspirated and replaced with fresh media before being incubated for up to 10 days, with a media change every other day. Cell patterns were observed on an hourly/daily basis using an inverted microscope equipped with a camera.

## Results

**Fig 2:**
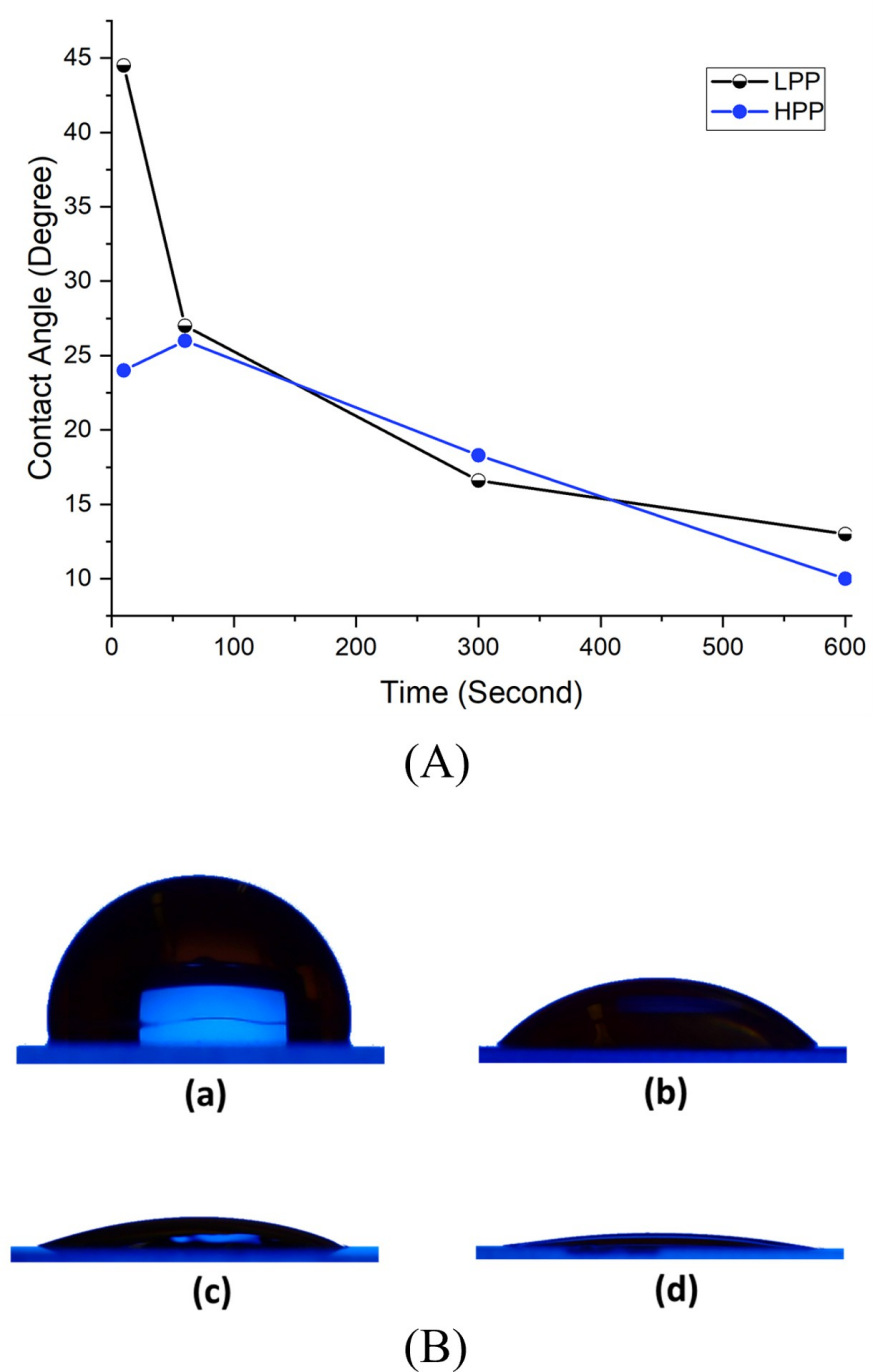
(A) Variation of DI water contact angle on plasma-treated COC surface for LPP and HPP at different exposure durations. (B) water droplets of the same volume on (a) untreated COC, and COC treated for (b) 10 seconds with LPP (c)10 seconds with HPP and (d) 10 minutes with HPP.

Plasma treatment is a well-known method for oxidizing some hydrophobic surfaces and converting them to hydrophilic ones. Several studies have used plasma to convert hydrophobic surfaces to hydrophilic ones [33,34]. Numerous factors, including the power of the plasma, the type of plasma, and the duration of the treatment, all affect the surface wettability following plasma treatment. This work examined the effect of two different plasma powers on surface wettability: 7.2 W (Low Power Plasma (LPP)) and 29.6 W (High Power Plasma (HPP)). In the current investigation, contact angle measurements on plasma-treated COC surfaces were made immediately after plasma treatment. The effect of varying the duration of LPP and HPP treatments on the water contact angle is illustrated in **Fig 2A**. COC with no treatment has a contact angle of 110 degrees, but HPP treatment reduces it to 24 degrees after only ten seconds of exposure and to 10 degrees after ten minutes. The LPP reduces the water contact angle to 45 degrees after ten seconds of exposure, and to 13 degrees after ten minutes. Our previous study examined the effect of plasma treatment on long-term exposure [32].

By adjusting the plasma power and duration of the treatment, it is possible to fine-tune the wettability of COC surfaces. **Fig 2B** illustrates images of four distinct drops of the same volume on the COC surface. The droplet in **Fig 2B part a** is on top of untreated COC. It has a contact angle of 110 degrees. In parts **b, c,** and **d** of the same figure, droplets with contact angles of 45, 24, and 10 degrees are shown on treated COC surfaces. For the three images (**b, c,** and **d**), the plasma treatment was 10 seconds LPP, 10 seconds HPP, and 10 minutes HPP, respectively.

**Fig 3:**
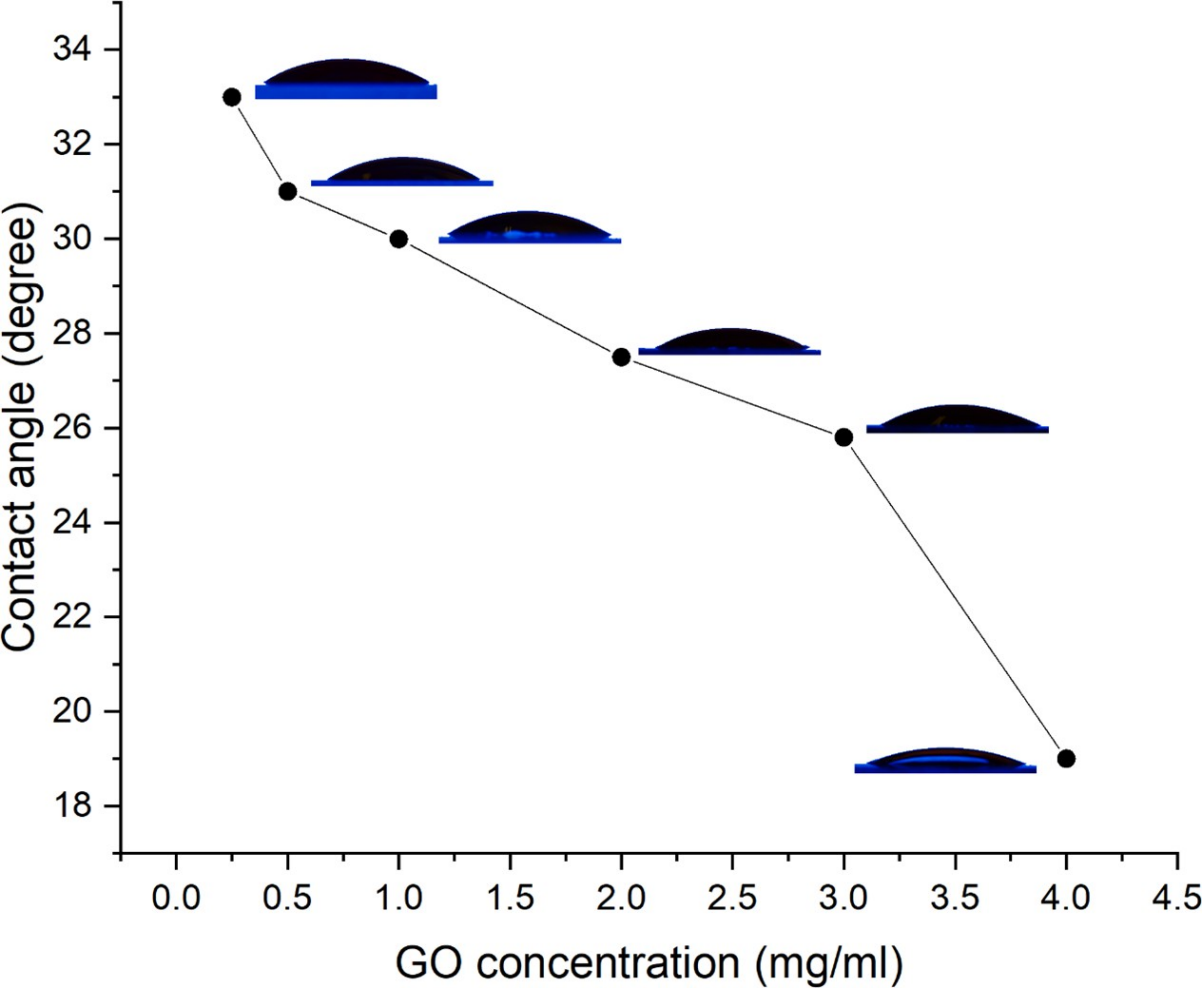
Variation of DI water contact angle on GO-coated COC surface at different GO concentrations.

Further investigation is conducted into the influence of GO concentration on the wettability of the COC surface. The current study examined the influence of GO concentrations of 0.25, 0.5, 1, 2, 3, and 4 mg/ml on wettability. All COC surfaces were treated in the same manner (5 minutes of HPP), but with varying concentrations of GO. **Fig 3** illustrates the effect of varying the GO concentration on the water contact angle. The figure clearly shows that increasing the concentration decreases the contact angle. DI’s contact angle with GO-coated COC is lowered from 33 degrees at 0.25 mg/ml to 19 degrees at 4 mg/ml GO concentration.

### Patterning the surface wettability of COC substrate

In this study, oxygen plasma treatment and GO-coating were used to change the surface wettability of the COC substrate and create hydrophilic patterned regions on a hydrophobic surface. The COC substrate is hydrophobic, with a water contact angle of 110° [19]. However, after five minutes of treating the COC substrate with oxygen HPP, the contact angle on the plasma-treated surface is reduced to 20° (**Fig 2**). This implies that oxygen plasma converts the COC’s hydrophobic surface to a hydrophilic surface, allowing cell growth.

More intriguingly, the wettability of the COC surface could be patterned to design specific hydrophilic shapes on the hydrophobic surface of the substrate using standard microfabrication techniques. These shapes will be the only hydrophilic areas of the substrate’s surface, with the rest of the surface being hydrophobic. This results in the formation of a heterogeneous surface, which can then be used to pattern cells on the surface, thereby enabling printing. The other method for patterning the surface wettability of the COC substrate is to deposit a thin film of GO, as illustrated in **Fig 1**. As shown in **Fig 3**, the water contact angle on the GO-coated COC surface is approximately 30° for GO concentration of 1 mg/ml. We previously investigated the chemical groups on COC, plasma treated COC, and GO-coated COC surfaces using FTIR spectra. The untreated COC substrate contains the C-H bending and stretching (aldehydic H and ethynenic H) modes. On the surface of pure GO and GO-coated COC, C = O and–OH groups are added to the surface. Additionally, plasma treatment introduces aldehydes and carbonyl groups; however, the intensity of these groups increases with GO modifications. This demonstrates that plasma treatment and GO coating of COC surfaces increase their hydrophilicity [32].

**Fig 4:**
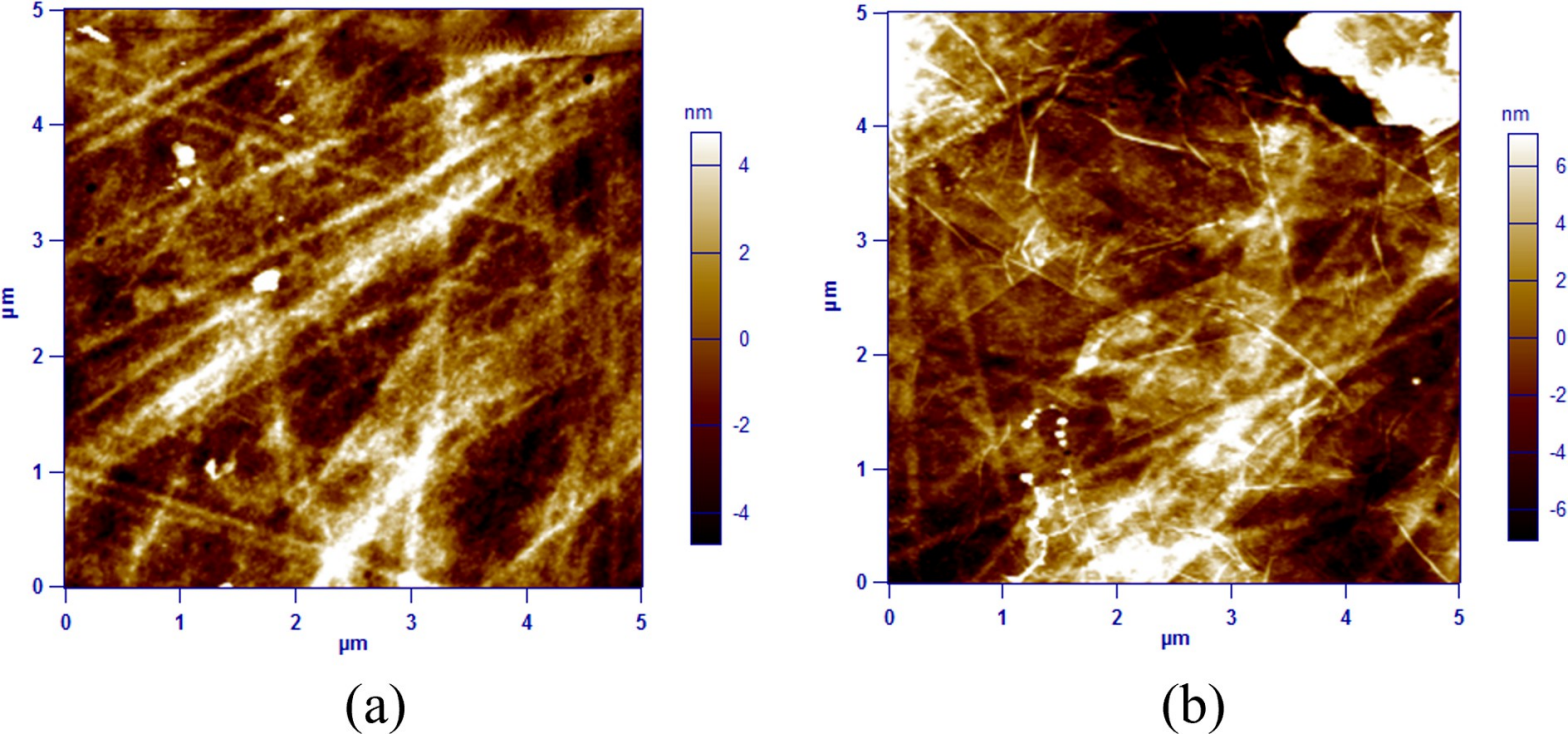
The AFM images for (a) COC plasma-treated surface and (b) GO-coated COC surface.

**Fig 5:**
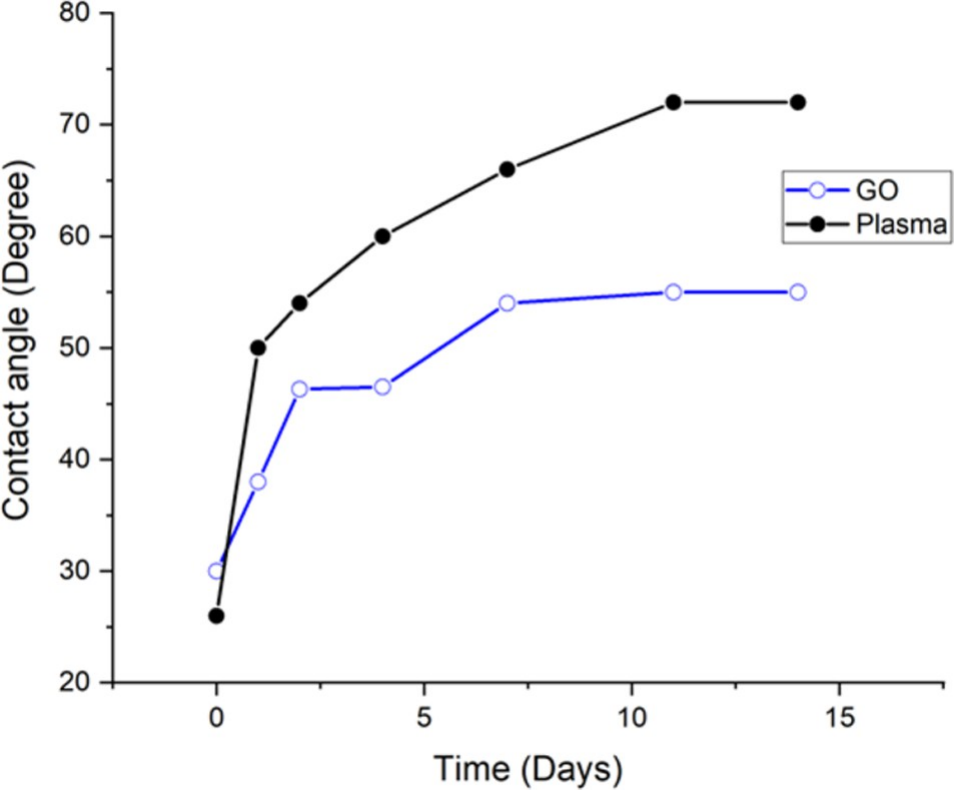
The change in water contact angle over time for plasma-treated (1 minute with HPP) and GO-coated COC substrates (1 mg/ml GO concentration). Both substrates were kept in dark area and at room temperature during the experiment.

Because surface roughness has an effect on a polymer’s or material’s wettability [1], the roughness of plasma-treated and GO-coated surfaces was determined using the AC mode topography of an Atomic Force Microscope (AFM), as illustrated in **Fig 4A and 4B**. The roughness of the GO-coated surface was determined to be 4.3 nm, while the surface treated with plasma had a roughness of 2.5 nm. Both surfaces were scanned over an area of 5μm x 5μm at a frequency of 1 Hz. Additionally, we investigated the effect of aging on the wettability of plasma-treated surfaces and GO-coated substrates by measuring the water contact angle immediately following fabrication and over time. As illustrated in **Fig 5**, the wettability of the surface was determined to be time-dependent using both techniques. On plasma-treated surfaces, the water contact angle increased from 20° immediately after treatment to around 50° after 24 hours. It has been reported that plasma-treated surfaces regain their hydrophobicity over time [35]. However, within the first 24 hours, there was no significant change in the wettability of the GO-coated surface. After 24 hours, the contact angle increased from 30° immediately after fabrication to 38°. The hydrophobicity of the surfaces became stable after nearly ten days of fabrication, and no significant change was observed afterwards. The water contact angle on the plasma-treated COC substrate reached a maximum value of 72°, whereas it did not exceed 55° on the GO-coated COC surface.

### Cell patterning on plasma-treated and GO-coated COC surfaces

**Fig 6:**
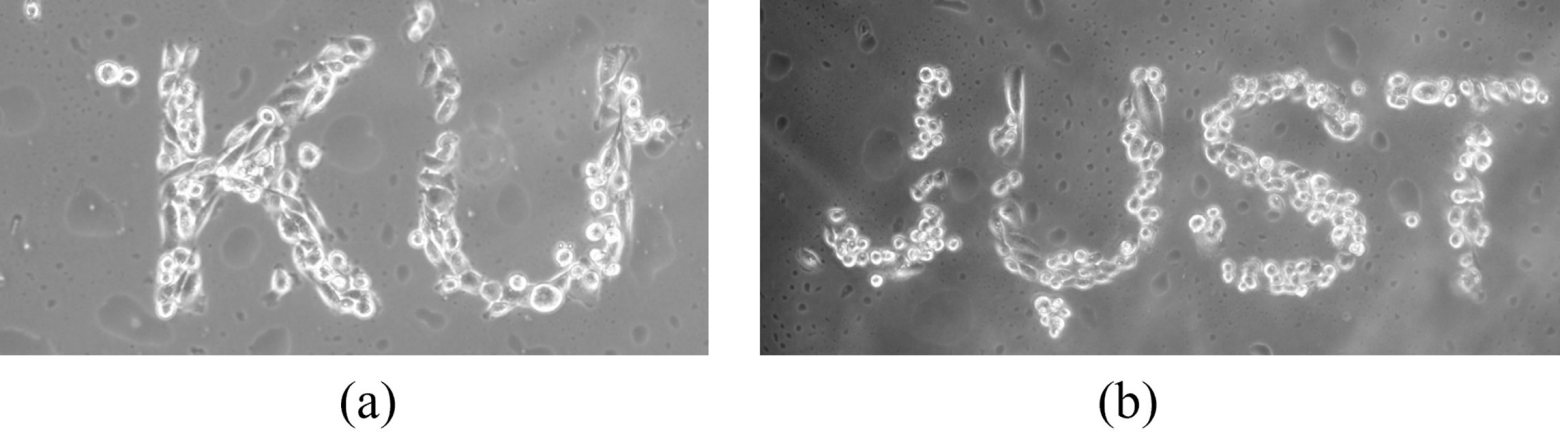
MDA-MB-231 cancer cells patterning in the shape of (a) KU and (b) JUST. The cells were cultured on the plasma-treated COC substrate in which the letters were made hydrophilic using oxygen plasma while the rest of the COC substrate was hydrophobic by nature.

Hydrophilic and charged surfaces promote cell adhesion and growth [29]. Plasma treatment improves cell adherence and growth by increasing the hydrophilic properties of various surfaces [11,13,36]. As a result, the plasma-treated patterned COC surface was used to investigate the impact of patterned wettability on cell attachment and growth. This hypothesis was tested using a breast cancer cell line (MDA-MB-231). Cells were seeded on a COC substrate with patterned wettability; the cells grew primarily on the plasma-treated areas of the COC substrate, with very few or no cells growing on the untreated areas. Furthermore, instead of expanding their growth on the COC surface, cells continued to grow on top of each other on the plasma patterned areas. Controlling cell growth on the surface enables printing using cells as the fundamental printing unit. Two microscopic images of MDA-MB-231 cells after seeding on a patterned plasma-treated COC substrate are shown in **Fig 6**. On top of the COC hydrophobic surface, the alphabet letters were treated with oxygen plasma to create hydrophilic surfaces. This wettability patterning for the COC surface modulated cell adherence and resulted in micropatterning of cell growth that can be used in cell and tissue culture fields.

**Fig 7:**
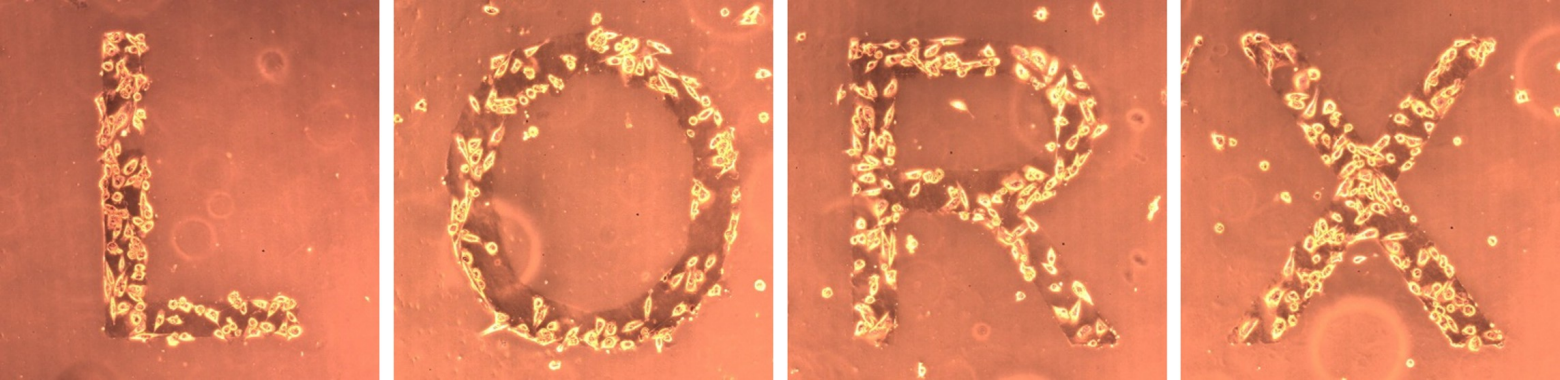
MDA-MB-231 cancer cells patterning in the shape of different alphabet letters. The cells were cultured on GO-coated COC substrates in which the letters were made hydrophilic using GO and the rest of the substrate was hydrophobic by nature.

Patterning the surface of COC with GO film not only improves the wettability over time but also increases the roughness of the patterned area **(Figs 4 and 5)**. As a result, patterned wettability using GO was investigated in order to pattern cell growth. Interestingly, the patterned GO surface drew cells to attach and grow on the GO hydrophilic surface **(Fig 7)**. The hydrophilicity of GO and the hydrophobicity of COC surfaces modulate cell adherence, resulting in cell growth micropatterning. Furthermore, instead of expanding their growth on the COC surface, the cells continued to grow over each other on GO patterned alphabetic letters. These novel cell micropatterning techniques will have a wide range of promising applications in cell and tissue culture.

**Fig 8:**
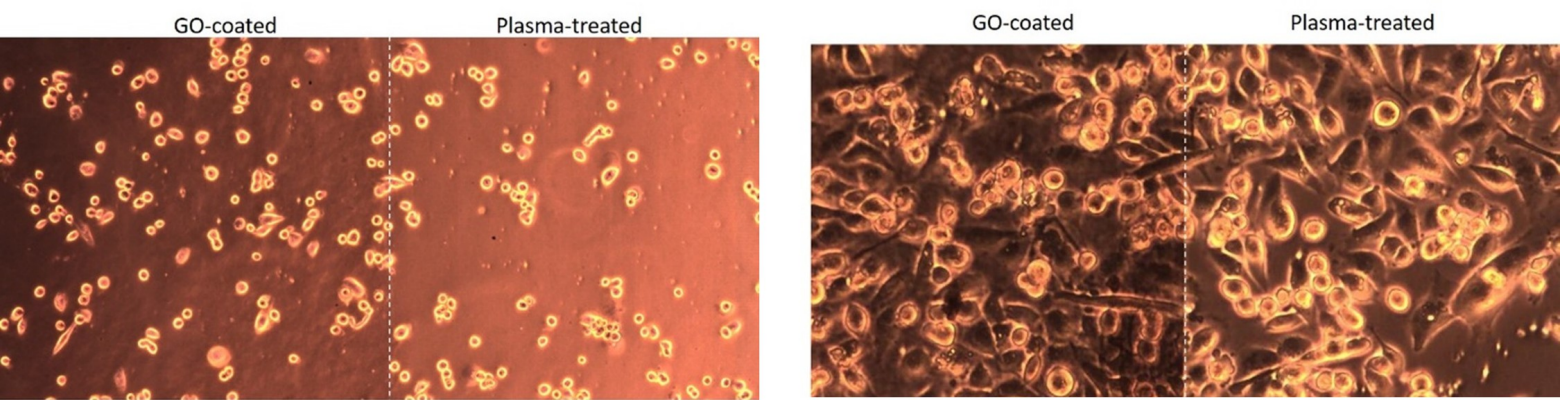
MDA-MB-231 cancer cells cultured on GO-coated and plasma-treated COC substrate (a) after 12 hours of seeding and (b) after five days of seeding.

To compare the effects of both techniques on cell patterning, cells were cultured on a substrate that had been patterned using both methods: plasma-treatment and GO-coating. **Fig 8A** shows a microscopic image of the cells after 12 hours of seeding, and **Fig 8B** shows the same after five days of seeding. The cells first acquired their elongated morphology on the GO-coated surface before acquiring it on the plasma-treated surface as shown in **Fig 8A**. This suggests that the cells adhere to the GO-coated surface prior to the plasma-treated surface. This could be due to the GO’s surface roughness when compared to the plasma-treated surface of the COC substrate. After a few days of seeding, cell growth on both surfaces appeared to be identical.

## Discussion

Surface properties, such as wettability, are important factors in determining cell biological behavior [37]. Surface wettability is a measure of surface energy that can be altered by the addition of hydrophilic or hydrophobic groups [38]. Tuning surface wettability is an important technique with numerous applications in various fields. Plasma treatment, for example, is a well-known procedure for increasing the hydrophilicity of many hydrophobic surfaces by incorporating different polar functional groups and modifying surface roughness [39]. Plasma treatment is accomplished through electrical discharges with various gases in a vacuum environment to achieve various effects on surface properties. Inert gases such as argon, reducing gases such as hydrogen, and reactive gases such as oxygen are examples of these gases [40]. Oxygen plasma has been shown to improve the wettability of many hydrophobic surfaces, including PDMS [41,42], PET polymer [42], and poly (lactide-co-glycolide) (PLGA) film [43]. To the best of our knowledge, this is the first study to focus on plasma-treated COC surfaces for cell micropatterning and the first study to print on polymer surface using cells through patterning the wettability. In this work, the surface wettability of COC material was tuned using oxygen plasma. According to our findings, plasma treatment reduced the contact angle of COC from 110° to 20° after 1 minute of exposure and to 10° after 10 minutes of exposure. This demonstrates that oxygen plasma added polar functional groups to the surface, making it more hydrophilic.

Plasma has been used to pattern cell growth [13,44]. Consistent with these findings, oxygen plasma treatment of the COC substrate was also successful in patterning cell growth. COC surfaces were patterned with plasma-printed alphabets. We investigated the possibility of using plasma-treated COC surfaces for cell culture because hydrophilic surfaces are more biocompatible with cell culture [29], and plasma treatment is a well-known procedure for making biocompatible surfaces for cell culture [11,43]. The results presented in this study revealed that the cells only attached and grew on the plasma-treated COC surface, with no cells growing on the native hydrophobic COC surface. This has the potential to be useful in a variety of fields, including cell and tissue culture.

On the other hand, oxygen plasma treatment does not maintain its effectiveness over time. The functional groups formed on oxygen plasma-treated surfaces are not long-lasting, as the surfaces’ hydrophilicity decreases over time [32,41,42]. In line with that, this study found that the contact angle of a plasma-treated COC surface drops to 20° immediately after one minute of treatment but then rises to more than 70° after a few days. As a result, a more stable material should be used to change the hydrophobicity of the COC surface. Previously, we demonstrated that GO is a stable material that can be patterned on a COC substrate using the plasma-enhanced lift-off technique [19]. The same technique was used in this study to test the ability of GO-patterned films to be used for cell patterning. First, the aging effect on the wettability of GO-coated COC surfaces was investigated, and the results revealed that the contact angle only slightly increases with time. Furthermore, when GO-coated surfaces were compared to plasma-treated surfaces, the surface roughness was found to be higher.

To investigate the possibility of using GO-coated COC surfaces to pattern cell growth, the wettability of GO-coated COC surfaces was first confirmed, followed by an investigation into the patterning of GO film into alphabets. The change in contact angle of COC surfaces after GO-coating implies improved wettability due to the deposition of a GO film. Furthermore, in this study, GO-coating was successfully used to pattern cell growth and print alphabetic letters using cells as printing unit, and the results were found to be comparable to those of the oxygen plasma treatment.

Finally, to test the biocompatibility of GO-coated COC surfaces, the same cells were cultured on GO-coated COC surfaces for up to ten days, and cell growth was monitored. Microscopic images reveal that cells grew exclusively on the GO-coated surface and not on the untreated hydrophobic COC surface. Even after the cells reached confluence, they preferred to grow on top of each other rather than spread to the untreated hydrophobic COC surface. Our findings support the ability of GO to pattern the surface of COC, which selectively improves the surface hydrophilicity and biocompatibility of the COC surface for cell growth and patterning. The reported method introduces a novel method for printing on COC polymer surface that utilizes living cells as the fundamental printing unit.

## Conclusion

Both oxygen plasma treatment and GO coating can be used to change the wettability of a COC substrate and create hydrophilic patterned regions on a hydrophobic surface. Both techniques, in turn, can modulate cell adherence and micropatterning of cell growth. The results presented in this paper show that when GO deposition is used to pattern the wettability of the COC surface, the surface roughness increases. Furthermore, the wettability changes for the GO-coated and plasma-treated COC surfaces were found to be time-dependent, with insignificant change for the GO-coated surfaces and a sudden significant change over time for the plasma-treated surfaces. Patterning the surface wettability of COC with GO has been found to be an easy, stable, and effective approach in cell micropatterning. The technique described here has very promising applications in the development of new cell assays and tissue culture. However, further research into the cellular-specific protein interaction to both surfaces, as well as the possibility of patterning different types of cells and tissues on these reported surfaces, is required.
